# Food Proteins and Peptides: Bioactivity, Applications and Health Benefits

**DOI:** 10.3390/foods15193399

**Published:** 2026-09-24

**Authors:** Ivan Cruz-Chamorro, Guillermo Santos-Sánchez

**Affiliations:** 1 Facultad de Enfermería, Universidad de Castilla-La Mancha, 02071 Albacete, Spain; 2 Université Paris-Saclay, AgroParisTech, INRAE, UMR PNCA, Palaiseau, France

## 1. Introduction

Food proteins are increasingly recognized not only as essential sources of dietary nitrogen and amino acids, but also as valuable precursors of peptides with a wide range of biological activities [1,2]. During enzymatic hydrolysis and gastrointestinal digestion, food proteins can release bioactive peptides capable of interacting with different physiological pathways and potentially contributing to the maintenance of human health [3]. Among the activities most extensively investigated are antioxidant, antihypertensive, anti-inflammatory, hypocholesterolemic, antidiabetic, and antimicrobial effects, highlighting the broad functional potential of protein-derived peptides [4,5,6,7].

The growing interest in these compounds is closely linked to the search for sustainable and naturally derived ingredients that can be incorporated into functional foods and nutraceuticals [8,9]. In this context, proteins from both conventional and alternative sources, including legumes, oilseed co-products, and other underutilized resources, are receiving increasing attention [10]. Advances in enzymatic hydrolysis, proteomic characterization, bioinformatics, and peptide identification have facilitated the discovery of new bioactive sequences and provided opportunities to improve the nutritional and functional value of food proteins. At the same time, the translation of these findings into practical applications requires consideration of peptide stability, bioaccessibility, gastrointestinal digestion, safety, and biological efficacy.

This Special Issue, entitled “Food Proteins and Peptides: Bioactivity, Applications and Health Benefits”, brings together recent research exploring the generation, characterization, biological activity, and potential applications of protein-derived peptides from diverse food sources. The contributions included in this collection address different aspects of this rapidly developing field, ranging from the production and characterization of protein hydrolysates to the evaluation of their biological effects and their incorporation into food or probiotic systems. Collectively, these studies illustrate the potential of food proteins and peptides as versatile ingredients for the development of innovative functional foods and nutraceuticals, while also highlighting the importance of further research to translate promising bioactivities into validated health applications.

## 2. Overview of the Published Articles

The five contributions included in this collection address different aspects of food protein and peptide research, covering a range of protein sources, biological activities, and experimental approaches. The studies examine silkworm, soybean, chickpea, rapeseed, and hempseed proteins, reflecting the diversity of materials currently being explored within this field. Their experimental strategies range from the enzymatic production and characterization of protein hydrolysates to proteomic and bioinformatic screening, animal studies, and evaluation in food matrices. The reported outcomes include effects on probiotic growth and stability, antihypertensive activity, antioxidant properties, and the identification of peptides with potential angiotensin-converting enzyme (ACE)- and dipeptidyl peptidase (DPP)-IV-inhibitory activities. Together, the contributions provide complementary perspectives on how protein-derived compounds can be investigated from their initial characterization and generation to the assessment of their biological properties and potential uses. The main objectives and key findings of each contribution are described below.

In **Contribution 1**, Huang et al. investigated soybean protein hydrolysates (SPHs) as an alternative nitrogen source for the cultivation and stabilization of probiotic Bifidobacterium strains. Enzymatic hydrolysis of soy protein isolate produced a hydrolysate rich in low-molecular-weight peptides, with 94.61% of peptides below 3 kDa. Increasing SPHs in the culture medium enhanced the growth of *Bifidobacterium breve* 1206, while experiments with free amino acids indicated that the effect was not solely attributable to amino acid availability, suggesting a role for specific peptides. SPH cultivation also induced changes in bacterial morphology and improved cell integrity and acid tolerance. Importantly, the use of SPH as the nitrogen source substantially increased survival during freeze-drying, particularly in *B. breve* 1206, with beneficial effects also observed in *Bifidobacterium longum* strains. Overall, the study highlights the potential of soybean protein hydrolysates as plant-derived nitrogen sources that can support probiotic growth while improving bacterial robustness during processing, offering promising applications in functional foods and probiotic formulations.

Anuduang et al. (**Contribution 2**) explored the potential of Thai silkworm (*Bombyx mori*) as an alternative source of protein and bioactive compounds by investigating hydrolysates obtained from three developmental stages: mature silkworm, A-silking silkworm, and pupae. The study first characterized their nutritional composition and amino acid profiles, revealing clear differences among developmental stages. Mature and A-silking silkworms showed higher protein contents, likely related to the presence of silk glands and their major proteins, fibroin and sericin, whereas pupae were characterized by a substantially higher fat content. Beyond their nutritional value, the authors investigated the potential health-related properties of the resulting hydrolysates, with particular emphasis on ACE-inhibitory and antioxidant activities. The mature silkworm hydrolysate showed the most pronounced ACE-inhibitory activity, and subsequent molecular-weight fractionation indicated that peptides below 3 kDa were particularly relevant to this activity. Notably, the ACE-inhibitory effect of this fraction reached 97.01%, approaching that of enalapril, used as a reference inhibitor. The high proportion of low-molecular-weight peptides identified in the mature silkworm hydrolysate further supports their potential contribution to its bioactivity. Conversely, the pupae hydrolysate exhibited the highest total phenolic content and the strongest antioxidant activity. These findings demonstrate that the bioactive potential of silkworm-derived hydrolysates varies according to developmental stage and composition. Overall, the study provides promising evidence for the valorization of different silkworm stages as alternative protein resources and highlights their potential to generate functional ingredients rich in bioactive peptides and antioxidant compounds, with possible applications in functional foods and nutraceutical products.

In **Contribution 3**, Figueroa-Salcido et al. investigated the potential of an optimized chickpea protein hydrolysate (OCPH) as a dietary strategy for the long-term management of hypertension, focusing on both its blood-pressure-lowering effects and the molecular mechanisms underlying this activity. Using spontaneously hypertensive rats, the authors evaluated the effects of daily OCPH supplementation for five weeks and monitored systolic, diastolic, and mean blood pressure, together with the expression of key components of the renin–angiotensin–aldosterone system in the kidneys.

OCPH produced a sustained antihypertensive effect, with reductions in systolic, diastolic, and mean blood pressure evident after only five days and maintained throughout the intervention. OCPH supplementation also reduced kidney weight, although further studies are needed to determine its significance. At the molecular level, OCPH increased renal expression of ACE2 and G protein-coupled Mas receptor (Mas1), whereas ACE1, renin, and angiotensin type 1 receptor (AT1R) remained unaffected. The negative association between ACE2 expression and blood pressure further supports the potential involvement of the ACE2/Ang-(1–7)/Mas1 axis, a counter-regulatory pathway of the renin–angiotensin system associated with vasodilatory effects.

Overall, this study provides evidence that chickpea protein hydrolysates may exert antihypertensive effects beyond direct ACE1 inhibition, potentially involving the vasodilatory ACE2/angiotensin-(1–7)/Mas1 pathway. Although further studies are needed to confirm the proposed mechanism, these findings highlight the potential of chickpea-derived bioactive peptides as promising ingredients for functional foods and nutraceuticals targeting cardiovascular health.

Garbacz et al. (**Contribution 4**) investigated the potential of rapeseed meal as a sustainable source of bioactive peptides by combining proteomic profiling with an in silico enzymatic hydrolysis. As a protein-rich co-product of oil extraction, rapeseed meal represents an attractive but still underexploited resource for plant-based functional ingredients. The authors characterized its protein profile using two-dimensional electrophoresis and MALDI-TOF/TOF mass spectrometry, identifying 50 protein spots, with cruciferin being the predominant protein, alongside several metabolic, defense-related, antioxidant, and oil-body-associated proteins.

The identified proteins were subsequently subjected to in silico hydrolysis using papain, ficin, and bromelain, and the resulting peptides were screened for potential biological activities using the BIOPEP-UWM database “https://biochemia.uwm.edu.pl/biopep-uwm/ (accessed on 16 September 2026)” and PeptideRanker “http://bioware.ucd.ie/~compass/biowareweb/Server_pages/help/peptideranker/help.php (accessed on 16 September 2026)”. Bromelain generated the highest number of predicted bioactive peptides, with 1592 peptides identified by BIOPEP, compared with 1424 and 1410 for papain and ficin, respectively. The predicted activities were particularly enriched in ACE- and DPP-IV-inhibitory and antioxidant peptides, while other potential activities included renin inhibition, antithrombotic, and hypolipidemic effects. Overall, 39 categories of biological activity were identified, highlighting the broad functional potential of rapeseed-derived peptides.

Importantly, these findings remain theoretical, as *in silico* digestion does not fully reproduce the conditions governing peptide release and activity in biological systems. Experimental hydrolysis, gastrointestinal digestion, peptide identification, and biological validation will therefore be required to confirm these predictions. Nevertheless, the study highlights rapeseed meal as a promising reservoir of potentially bioactive peptides and demonstrates how proteomics and bioinformatics can be used to prioritize proteins, enzymes, and peptide sequences for further investigation. These findings support the potential of rapeseed-derived proteins as sustainable ingredients for future functional food and nutraceutical applications.

Finally, in the last contribution (**Contribution 5**), Angeletti et al. investigated the potential of hempseed protein hydrolysates as natural antioxidants in meat products, extending previous evidence of their antioxidant activity in screening assays and oil-in-water nanoemulsion systems to a more complex and practical food matrix. The study evaluated two Alcalase-generated hempseed hydrolysates, obtained from hempseed meal (AHM10) and hempseed protein isolate (AHPI10), which were incorporated at 0.4% into beef and turkey meatballs and compared with a synthetic antioxidant, EDTA, at 100 ppm. Lipid oxidation was monitored during 14 days of refrigerated storage at 4 °C using peroxide value (PV) and thiobarbituric acid reactive substances (TBARSs), representing primary and secondary oxidation products, respectively. Among the treatments, AHM10 showed the strongest protective effect in beef meatballs, maintaining peroxide values below 5.0 meq hydroperoxides/kg oil and TBARSs below 1.8 mg MDA/kg oil throughout storage. Notably, AHM10 performed better than EDTA in the beef system, suggesting a pronounced ability to inhibit both the formation and progression of lipid oxidation, potentially related to the metal-chelating and antioxidant properties previously reported for this hydrolysate. In turkey meatballs, however, the antioxidant effects were less pronounced, and neither hempseed hydrolysate outperformed EDTA in terms of secondary lipid oxidation, highlighting the influence of the food matrix on hydrolysate functionality. Importantly, the incorporation of hempseed hydrolysates did not result in major sensory disadvantages. Consumer acceptance testing after 14 days showed no significant differences among treatments for the main acceptability attributes, while AHM10-containing beef meatballs had the lowest proportion of consumers detecting an off-flavor and the highest numerical percentage of consumers willing to eat the product again. Overall, the findings demonstrate that hempseed-derived protein hydrolysates, particularly AHM10, can retain meaningful antioxidant functionality when incorporated into complex food systems and may offer a promising natural alternative to synthetic antioxidants. The study also highlights the importance of evaluating both oxidative stability and sensory performance when assessing the potential of plant protein hydrolysates as functional food ingredients.

## 3. Conclusions

This Special Issue provides valuable insights into the potential of protein hydrolysates and bioactive peptides as multifunctional ingredients for food, nutrition, and health-related applications. Across the five contributions, several complementary themes emerge, including the valorization of alternative protein sources, the identification of bioactive peptides, and the evaluation of antioxidant, antihypertensive, probiotic-supporting, and food-preserving properties.

Collectively, these studies highlight the versatility of protein hydrolysates as promising ingredients for sustainable and health-oriented food innovation. We hope that the contributions presented in this collection will encourage further multidisciplinary research integrating food science, nutrition, protein chemistry, and microbiology, ultimately supporting the development of innovative and scientifically validated protein-based ingredients and food products.
